# Neurological conditions among pediatric patients seeking care at a tertiary level hospital in western Kenya

**DOI:** 10.1371/journal.pone.0321501

**Published:** 2025-04-15

**Authors:** Eren Oyungu, Megan Song McHenry, Chrispine Oduor, Jamil Said, O’Brien Kyololo, Jane R. von Gaudecker

**Affiliations:** 1 Moi University, Department of Medical Physiology, Eldoret, Kenya; 2 Indiana University School of Medicine, Indianapolis, United States of America; 3 Moi University, Department of Medicine, Eldoret, Kenya; 4 Moi University, Department of Human Anatomy, Eldoret, Kenya; 5 Moi University School of Nursing & Midwifery, Eldoret, Kenya; 6 Indiana University School of Nursing, Indianapolis, United States of America; Duke University Medical Center: Duke University Hospital, United States of America

## Abstract

Neurological disorders significantly contribute to the global burden of diseases, especially in sub-Saharan Africa. However, within local contexts in Kenya, little is known about the pattern of neurological and neurosurgical disorders within pediatric populations. A 3-month cross-sectional observational study was conducted at a tertiary-level hospital in western Kenya to describe these patterns and basic characteristics of the patient population. Consecutive pediatric patients presenting for neurological and neurosurgical care in inpatient and outpatient settings at Moi Teaching and Referral Hospital were included in the study. A total of 485 patients were included in the study. The average age of the patients was 6.2 years, and most were male (57%). Out of these, 30.3% (n=147) were inpatients, and 69.7% (n=338) were outpatients. Inpatients traveled longer distances compared to outpatients (P<0.001), and most were from rural areas (P<0.0001), while outpatients were mostly from peri-urban areas (P<0.0001). The majority of the inpatients (25%) and outpatients (69%) had a diagnosis of epilepsy. Other common neurological conditions were neurodevelopmental delays and meningitis/encephalitis. Given the burden of these diseases, there is a need to improve the health infrastructure for better access to quality healthcare. Specifically, improving epilepsy care, supporting neurodevelopmental programs, managing infectious diseases, and expanding neurosurgical services can enhance health infrastructure for this population.

## Introduction

Neurological and neurosurgical disorders in pediatric patients present significant challenges, often affecting a child’s physical, cognitive, and emotional health and contributing to increased morbidity and mortality [1]. These conditions frequently result in long-term challenges in education, social integration, and overall quality of life, creating a burden not only on children and their families but also on healthcare systems [2]. Neurological disorders contribute a significant proportion of the global burden of disease, especially in sub-Saharan Africa (SSA) [3], where resources for diagnosis and management are often limited.

In SSA, epilepsy and neurodevelopmental delays are among the most prevalent pediatric neurological conditions. SSA hosts the majority of the world’s epilepsy cases, with a prevalence of 5–10 per 1,000 children, largely due to preventable causes like infections and birth complications [4]. Developmental disorders such as cerebral palsy have a prevalence of 1.5 to 2.5 per 1,000 live births [5]. A recent systematic review found that developmental delays are prevalent in low—and middle-income countries at 18.83%, while studies specific to sub-Saharan Africa indicated a higher prevalence of 26.69% [6].

Despite its high prevalence, most data on pediatric neurological conditions relies on population-based, epidemiological studies [3,7]. Few hospital-based studies exist and little is known about the patterns of general neurological and neurosurgical disorders seen among pediatric patients within regional settings [8]. Understanding patient demographics and trends within a healthcare setting can provide valuable insights for advocacy, strengthen the healthcare system, and assist in the planning and implementation of effective interventions. Furthermore, identifying these patterns can inform public health initiatives and resource allocation, ultimately improving healthcare outcomes in the region. Very little has been reported on hospital-based epidemiological data involving the pediatric population with neurological or neurosurgical conditions in Kenya, which are key data needed to help guide program development. Therefore, the purpose of this study was to describe the patterns of neurological and neurosurgical disorders among pediatric patients at a tertiary-level academic hospital in western Kenya. By understanding the patterns and burden of neurological diseases, we may tailor future care and services to the needs of the community.

## Methods

Setting: This cross-sectional observational study was conducted at the Moi Teaching and Referral Hospital (MTRH), the second-largest national referral and teaching hospital in Kenya. The hospital serves a population of 24 million people throughout western Kenya, parts of eastern Uganda, and southern Sudan. The Shoe4Africa Children’s Hospital, the 2^nd^ free-standing public children’s hospital in SSA, is part of MTRH. It has 200–250 inpatients daily and, along with MTRH, provides care to more than 400,000 inpatients and outpatients annually. MTRH and Moi University have long-term partnerships with a group of North American institutions collectively known as AMPATH (Academic Model Providing Access to Healthcare) consortium. At the time of this study, one Kenyan pediatric neurologist (EO) attended to neurology consults in the wards and conducted weekly outpatient neurology clinics. Visiting neurologists are routinely seconded by the AMPATH consortium to support local personnel. There are six neurosurgeons on staff. The resources available at MTRH for neurology care include: one MRI, two CT scanners, one EEG machine and a laboratory with capability for cerebrospinal fluid investigations.

The study was approved as an exempt study by the Institutional Review Board at Indiana University, USA (IRB # 1811345313) and the Institutional Research Ethics Board at Moi University, Kenya (IREC approval # 003160). For inpatients, parents/guardians were contacted to be informed about the study within 24 hours of admission. For outpatients, the patient’s parent/guardian attending the neurology clinic on their respective clinic days were contacted after they were seen by the healthcare provider. Verbal informed consent and assent were obtained from parents/guardians and children before data collection. This verbal approval was marked as the first item in each participant’s data collection tool.

Study participants: Inclusion criteria for this study were: (1) the patient was ≤18 years of age, (2) seeking care at MTRH within either inpatient or outpatient settings, (2) must have a guardian or caregiver present, (3) had a primary diagnosis or a neurological or neurosurgical condition (henceforth known only as a neurological condition). No patients declined to participate. Given that the purpose of this study was to gain insights into the demographic details of all patients with neurological or neurosurgical disorders attending MTRH, there were no exclusion criteria applied.

Data collection: This 3-month cross-sectional observational study was conducted between March 1, 2019, to May 31, 2019, where pediatric patients seeking neurological care were prospectively identified within inpatient and outpatient settings. Data were collected by three research assistants (RAs) who had clinical background and collaborative institutional training initiatives (CITI) trained. Prior to data collection, the RAs received training in participant identification, recruitment, and REDCap data entry. During the study period, the RAs conducted daily reviews of hospital admission records and inquiries in the wards where these patients were likely to be admitted. Data were collected from patients’ records and parent/guardian inquiries.

Variables: The authors designed the survey based on the neurological diagnoses utilized for medical documentation at MTRH. The disease categories seen in both inpatients and outpatients include: “neurodegenerative disease” included presumed encephalitis, meningitis and encephalopathy, “cerebrovascular disease” includes hemorrhagic and Ischemic stroke, “developmental disorders” included congenital brain anomalies, and other developmental disorders. The RA’s abstracted data from patient files, the diagnosis on file provided by the primary care team was used to identify the cases – the authors did not perform further adjudications of cases identified. The age category was divided by infants (0–1.99 years), pre-primary (2.00–4.99 years), primary (5.00–13.99 years) and school age (14–18 years).

Statistical Analyses*:* The Wilcoxon rank sum test was used to test for differences between inpatient and outpatient continuous variables, and a two-sample t-test or chi-square test was used to compare the distributions of percentages of categorical variables between inpatient and outpatient visits. The significance level was set at 0.05.

## Results

A total of 485 pediatric patients seeking treatment for neurological conditions were included in the study. Out of these, 30.3% (n=147) were inpatients and 69.7% (n=338) were outpatients. The mean and standard deviation (SD) of the age of children 6.2 (4.4) years. Nearly half (46.8%, n=227) were > 5 years in age. Approximately a third of outpatients were in the 2–5 years age group (n=116, 34.3%), and nearly half of the outpatients were in the 5–14 years age group (n=70; 47.6%). Compared to outpatients, the inpatients traveled longer distances from home to MTRH (median: 50 vs. 12 kilometers; (P<0.001). A more significant percentage of inpatients were from rural areas (n= 146; 43.2%; *P*<0.0001) and outpatients from peri-urban areas (n= 192; 56.8%; *P*<0.0001). Demographic details are provided in Table 1.

**Table 1:** Demographic details of inpatients and outpatients.

Variables		Inpatient	Outpatient	P-value
		147 (30.3%)	338 (69.7%)	
Age	Mean ± SD	5.7 ±4.3	6.4 ±4.4	0.1223
	0-1.99 years	26 (17.7)	39 (11.5)	0.1045
		2.00-4.99 years	46 (31.3)	116 (34.3)
		5.00-13.99 years	70 (47.6)	157 (46.4)
		14-18 years	5 (3.4)	26 (7.7)
Gender	Male	83 (56.5)	193 (57.1)	0.8962
	Female	64 (43.5	145 (42.9)	
~ distance from home to MTRH (Kilometers)	Mean (SD)	81.8 (73.6)	41.3 (72.8)	<.0001
	Median (IQR)	50.0 (15.0, 130.0)	12.0 (4.1, 45.6)	
Residence	Rural	101 (68.7)	146 (43.2)	<.0001
	Periurban	46 (31.3)	192 (56.8)	

**Fig 1:**
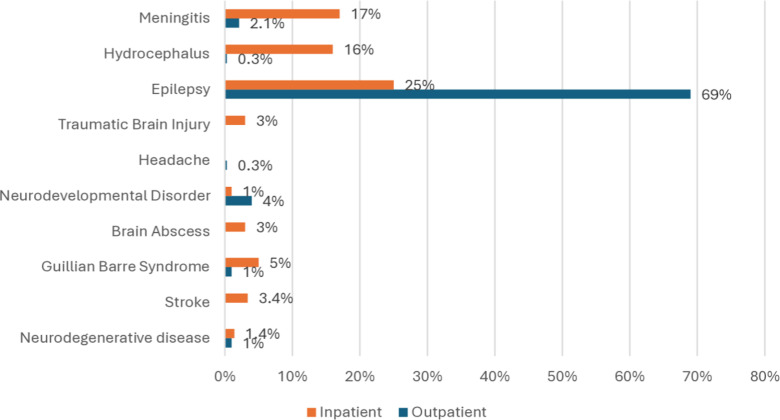
Major pediatric diagnoses.

Diagnoses: Fig 1 (major pediatric diagnoses) shows the primary neurological diagnoses for all pediatric inpatients and outpatients at MTRH. Most outpatients (69%) and inpatients (25%) had a diagnosis of epilepsy.

Of the 485 pediatric patients, 101 had multiple comorbid neurological conditions. For example, 37 inpatients had diagnoses of epilepsy and neurodevelopmental disorders; 5 inpatients had diagnoses of epilepsy and brain abscess; and seven outpatients had diagnoses of headache and epilepsy. No significant differences were found in the ages of inpatients and outpatients. Overall, the average length of stay for pediatric inpatients was 13.5 days (SD = 14.1; median= 9). Table 2 details the age and length of stay of patients with major neurological diagnoses.

**Table 2:** Age and Length of stay of patients with major neurological diagnoses.

Disease condition		Age			Inpatient length of stay (in days)
		Inpatient	Outpatient	P-value	
Meningitis/encephalitis	**N**	**52**	**30**		**44**
	Median (IQR)	3.5 (1.0, 6)	3.0 (1.5, 5.5)	0.0771	9.0 (6.0, 21.5)
Cerebrovascular disease	**N**	**6**			**6**
	Median (IQR)	9.5 (2.0, 11.0)			10.0 (6.0, 15.0)
Gulliane Barre Syndrome	**N**	**7**	**2**		**7**
	Median (IQR)	11.0 (5.0, 12.0)	9.0 (4.0, 14.0)	0.9999	9.0 (4.0, 12.0)
Brain Abscess	**N**	**12**	**3**		**12**
	Median (IQR)	9.5 (6.5, 12.0)	10.0 (1.0, 14.0)	0.9999	13.0 (6.5, 18.5)
Neurodevelopmental disorders	**N**	**1**	**50**		**1**
	Median (IQR)	6.0 (6.0, 6.0)	3.0 (2.0, 4.0)	0.2641	12.0 (12.0, 12.0)
Headache	**N**		**8**		**0**
	Median (IQR)		12.5 (11.0, 14.0)		
Traumatic Brain Injury	**N**	**5**			**5**
	Median (IQR)	11.0 (10.0, 13.0)			5.0 (2.0, 6.0)
Epilepsy	**N**	**46**	**301**		**46**
	Median (IQR)	4.0 (2.0, 11.0)	5.0 (3.0, 11.0)	0.0747	8.5 (5.0, 17.0)
Hydrocephalus	**N**	**38**	**6**		**38**
	Median (IQR)	3.0 (2.0, 6.0)	1.0 (1.0, 2.0)	0.0752	9.0 (5.0, 16.0)

## Discussion

This study provided important information regarding the pediatric patients being evaluated and managed for neurological conditions in a tertiary academic hospital within a 3-month period. Consistent with the population-based prevalence studies from Africa, epilepsy was found to be one of the top conditions for both inpatient and outpatient visits. Encephalitis/meningitis was the second most common diagnosis among the inpatients and contributed to long stays in the hospital/bed occupancy [9,10]. Hydrocephalus was the most common neurosurgical condition seen among inpatients. Understanding the pediatric neurological conditions seen most commonly within this setting is an important first step in optimizing services and meeting patient care needs.

Over 85% of the global burden of epilepsy occurs in low and middle-income countries, with a majority of these in Africa [9]. Within SSA, prior infectious and perinatal insults are the most common etiologies of epilepsy. Nearly 75% of these children are likely to have a neurodevelopmental delay [10]. Within our study, epilepsy and neurodevelopmental delays were both common diagnoses, both individually and as co-morbid conditions. For example, a child with epilepsy would also present with developmental delays. Children with these conditions do more hospital visits and are on long-term medications that require regular monitoring, and in many settings globally, the treatment gap for optimal neurological care is great [11,12]. Studies like this are an important first step in understanding the needs of the population in low-resourced settings.

Meningitis and encephalitis were among the most common neurological conditions diagnosed in children. Globally, deaths from meningitis have increased by over 20% since 1990 and there were over 2.8 million cases of meningitis globally in 2016 [13]. Infections of the central nervous system are strongly associated with the development of epilepsy, especially with infections occurring in the neonatal period [14]. Prevention and early management of meningitis and encephalitis can reduce the risk for further neurological sequelae and should be a priority for health care settings in low-and middle-income settings.

Our study also noted that individuals with neurological conditions requiring inpatient services lived further away from MTRH than those who were outpatients. This is similar to adult population who seek care for neurological conditions in the same hospital [15]. With Shoe4Africa being the only public, freestanding children’s hospital in Kenya, it is not surprising that t families were willing to travel far distances to seek care for severely sick children. However, evidence of this is important when considering follow-up instructions and care for neurological conditions that will require long-term management, such as epilepsy. Healthcare teams should consider providing patients with clear follow-up instructions before hospital discharge to ensure optimal health outcomes.

This cross-sectional study is the first of its kind in Western Kenya to provide a detailed description of the characteristics of pediatric patients with neurologic and neurosurgical conditions seen at one of only two tertiary referral hospitals in the country. This study has some limitations to consider. Because we did not have the numbers of the inpatient and outpatient pediatric visits during the 3-month time period, it was difficult to calculate point prevalence for pediatric neurological conditions within this setting. Additionally, there may have been variations related to patient volumes over the course of the year. However, there were no social or environmental perturbations during the period that would suggest the given time frame would be subject to specific bias. This study intentionally excluded details about various co-morbid conditions associated with the primary condition to maintain a focused and streamlined analysis. Including such information could complicate the study’s objectives. Additionally, the reasons for referrals to the hospital were not addressed, as they were deemed outside the study’s primary scope; doing so would require a broader investigation into healthcare systems and referral practices. By concentrating on the main subject, the study aims to provide clear and concise insights that can serve as a foundation for future research in these other important areas.

## Conclusion

In summary, this study provides useful information about the frequency and categorization of specific neurological conditions seen in children within MTRH. Overall, epilepsy was the most common condition in both outpatients and inpatients. Among outpatients, neurodevelopmental disorders were the second most common category. Encephalitis/meningitis was the second most common neurological diagnosis among inpatients, while hydrocephalus was the most common neurosurgical diagnosis. One pediatrician was responsible for seeing all children with neurological conditions and a regular pediatric clinical load. This highlights the high burden of disease and the need for additional infrastructure to support access to services and quality care for this population. The findings of this study provide valuable insights to inform future care and services for pediatric patients in Kenya.

## Supporting information

S1 File Study intake form. (DOCX)

## Notes

Each author has contributed substantially to this work.
